# An autosomal recessive variant in *PYGM* causes myophosphorylase deficiency in Red Angus composite cattle

**DOI:** 10.1186/s12864-024-10330-1

**Published:** 2024-04-27

**Authors:** Mackenzie C. Batt, Leila G. Venzor, Keri Gardner, Rachel R. Reith, Kelsey A. Roberts, Nicolas J. Herrera, Anna M. Fuller, Gary A. Sullivan, J. Travis Mulliniks, Matthew L. Spangler, Stephanie J. Valberg, David J. Steffen, Jessica L. Petersen

**Affiliations:** 1 Department of Animal Science, University of Nebraska-Lincoln, Lincoln, NE USA; 2 College of Veterinary Medicine, Michigan State University, East Lansing, MI USA; 3 School of Veterinary Medicine and Biomedical Sciences, University of Nebraska-Lincoln, Lincoln, NE USA

**Keywords:** *Bos taurus*, Glycogen storage disease type V, Exercise intolerance, De novo variant, Livestock production, Mendelian disease, McArdle disease, Dark cutter

## Abstract

**Background:**

Between 2020 and 2022, eight calves in a Nebraska herd (composite Simmental, Red Angus, Gelbvieh) displayed exercise intolerance during forced activity. In some cases, the calves collapsed and did not recover. Available sire pedigrees contained a paternal ancestor within 2–4 generations in all affected calves. Pedigrees of the calves’ dams were unavailable, however, the cows were ranch-raised and retained from prior breeding seasons, where bulls used for breeding occasionally had a common ancestor. Therefore, it was hypothesized that a de novo autosomal recessive variant was causative of exercise intolerance in these calves.

**Results:**

A genome-wide association analysis utilizing SNP data from 6 affected calves and 715 herd mates, followed by whole-genome sequencing of 2 affected calves led to the identification of a variant in the gene *PYGM* (BTA29:g.42989581G > A). The variant, confirmed to be present in the skeletal muscle transcriptome, was predicted to produce a premature stop codon (p.Arg650*). The protein product of *PYGM*, myophosphorylase, breaks down glycogen in skeletal muscle. Glycogen concentrations were fluorometrically assayed as glucose residues demonstrating significantly elevated glycogen concentrations in affected calves compared to cattle carrying the variant and to wild-type controls. The absence of the *PYGM* protein product in skeletal muscle was confirmed by immunohistochemistry and label-free quantitative proteomics analysis; muscle degeneration was confirmed in biopsy and necropsy samples. Elevated skeletal muscle glycogen persisted after harvest, resulting in a high pH and dark-cutting beef, which is negatively perceived by consumers and results in an economic loss to the industry. Carriers of the variant did not exhibit differences in meat quality or any measures of animal well-being.

**Conclusions:**

Myophosphorylase deficiency poses welfare concerns for affected animals and negatively impacts the final product. The association of the recessive genotype with dark-cutting beef further demonstrates the importance of genetics to not only animal health but to the quality of their product. Although cattle heterozygous for the variant may not immediately affect the beef industry, identifying carriers will enable selection and breeding strategies to prevent the production of affected calves.

**Supplementary Information:**

The online version contains supplementary material available at 10.1186/s12864-024-10330-1.

## Background

Optimizing muscle growth and function is crucial to success in beef production. Disruption of muscle metabolism can be both detrimental to the product and a concern for animal welfare. Numerous musculoskeletal conditions influence the efficiency of beef cattle production. Examples of genetic myopathies include a variant in *MSTN* (myostatin) that can lead to muscle hypertrophy, which can be desirable for production but can cause dystocia [1]. Lipomatous muscular dystrophy identified in Piedmontese cattle results in muscle tissue degeneration and fatty infiltration, causing weakness and poor meat quality [2]. In Charolais cattle, a recessive variant in *PYGM* (glycogen phosphorylase; muscle associated) causes myophosphorylase deficiency, leading to exercise-induced rhabdomyolysis and recumbency, impacting animal well-being [3]. These examples underscore the importance of genetic management in cattle breeding to balance optimal muscle development and animal welfare.

**Fig. 1:**
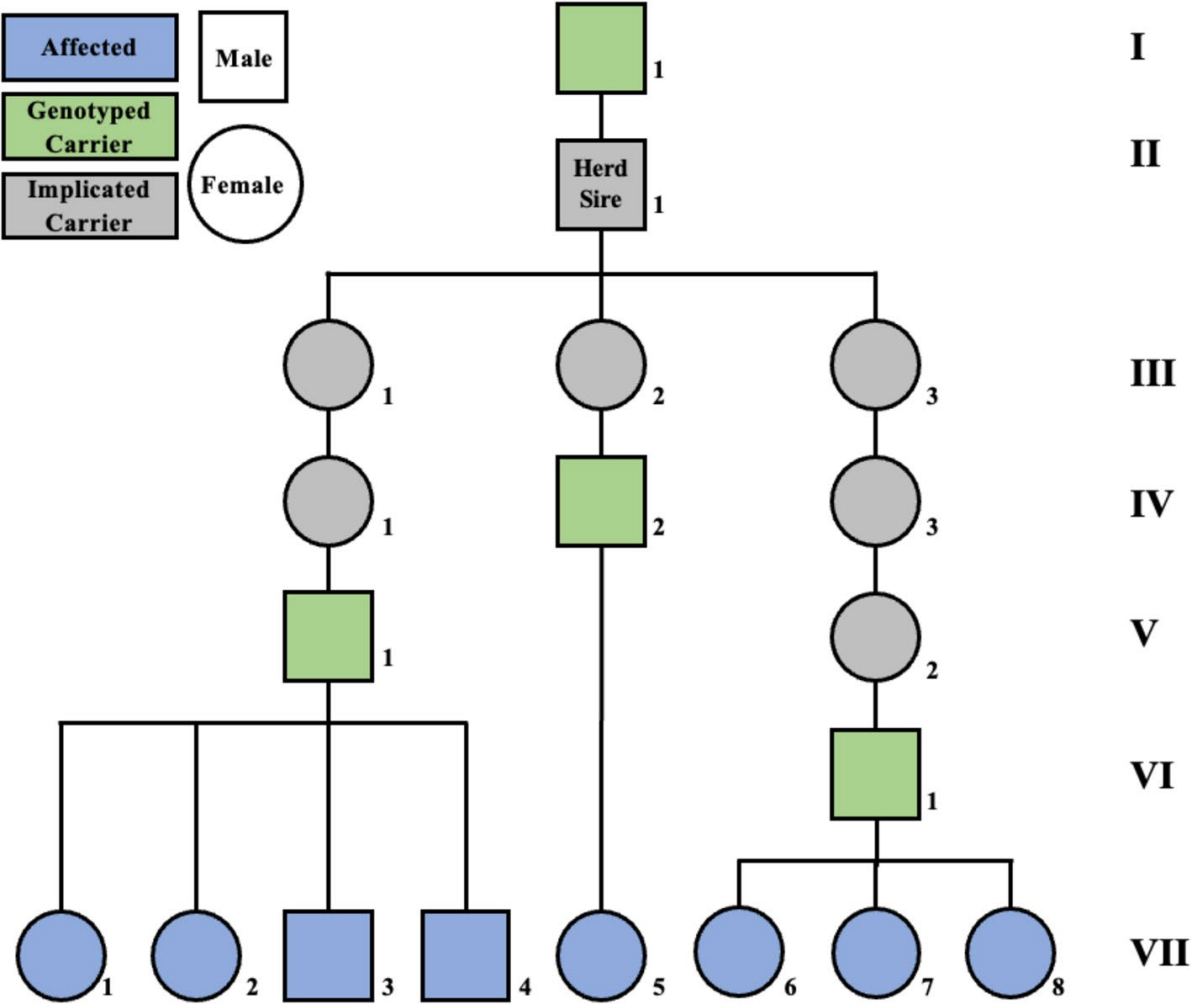
Pedigree of affected calves. Two were sold (VII-1,5), two died in the pasture (VII-2,3), one was euthanized (VII-4), and the remaining calves are denoted as: Calf A (VII-6), Calf B (VII-7), and Calf C (VII-8)

Composite (Red Angus, Simmental, Gelbvieh) calves from a Nebraska herd presented with exercise intolerance exacerbated by stress (Additional File 1). Initially, affected calves (1–6 months of age) were observed lagging behind the herd when moving between pastures. Under conditions requiring the calves to move at a more accelerated pace, calves would reportedly collapse and remain recumbent for brief periods of time. The calves had no known Charolais ancestry and tested negative for the previously identified *PYGM* variant. The sires of the affected calves (*N* = 3) had a common paternal ancestor (Fig. 1); pedigrees were unavailable for the dams (*N* = 8) although heifers were retained as replacements within the herd and were sometimes mated to related bulls. Therefore, it was hypothesized that a de novo autosomal recessive variant was causative of exercise intolerance in the affected calves. Genome-wide single-nucleotide polymorphism (SNP) data and whole-genome sequencing (WGS), followed by functional analyses of skeletal muscle from affected cattle and unaffected herd mates, were employed to test this hypothesis. Identifying a causative variant would enable breeders to make informed breeding decisions to manage this variant within the population effectively.

## Methods

### Affected calves

Two affected calves, Calf A (7 months) and Calf B (5 months), were transported from the ranch at which they were born to the University of Nebraska-Lincoln (UNL) for biopsies and clinical observation before being moved to UNL Eastern Nebraska Research, Extension and Education Center (ENREEC) feedlot (Mead, NE). Both calves (heifers) exhibited exercise intolerance on the ranch. At approximately one year of age, while in the feedlot, Calf A collapsed and experienced tremors that resembled seizure-like signs. This event resulted in a decision to euthanize to establish a diagnosis. Calf B (heifer) reached market weight (462 kg) at ENREEC prior to being harvested at the USDA-inspected Loeffel Meat Laboratory at UNL.

Calf C (heifer) presented with similar clinical signs and was housed at the UNL Animal Science Complex until harvest at the Loeffel Meat Laboratory at 14.5 months (310 kg). Five other affected calves on the ranch were not transported live to UNL. Two of those calves (E and F) were found deceased in the pasture (3 and 4 months of age) and one calf (D) was euthanized after it went down and could not get back up when pressured to move (3.5 months of age). The other two calves (G and H) were sold after being transported to the UNL West Central Research and Extension Center Feedlot (North Platte, NE).

### Sample collection

DNA samples in the form of whole (EDTA) blood (*N* = 33), tissue (*N* = 2), TSUs (*N* = 10), blood cards (*N* = 320), semen (*N* = 9), and hair (*N* = 7) were collected from a total of 381 animals.

Calf A, Calf B, and later, five additional animals from the herd (chosen to represent both carriers and controls of the candidate genotype, see below) underwent muscle biopsies. During the procedure, 6 ml of 2% lidocaine (20 mg/ml) was injected subcutaneously over the *semimembranosus* muscle. A 5 cm incision was made to retrieve the muscle, and the skin was sutured to close the incision. The collected samples were flash-frozen using liquid nitrogen and stored at -80 °C.

Blood serum samples were collected from three affected calves (A, B, and C) before and 3 h after the cattle were worked to simulate exercise (moved up and down an alleyway or in an arena for approximately 45 min).

### Histology and blood profile

Sections of muscle biopsy fixed in 10% neutral buffered formalin were processed as routinely conducted [4] for hematoxylin and eosin and Periodic acid-Schiff (PAS) stains in an AAVLD-accredited laboratory. A complete blood count and serum chemistry analysis was conducted on the three affected calves before and after exercise. The assays were carried out by a CLIA-certified commercial laboratory (Physicians Laboratory Inc, Lincoln, NE).

### Pedigree analysis

Pedigrees of animals in the herd were recorded and queried from the American Simmental Association database [5]. The pedigrees of Red Angus animals were sourced from the Red Angus Association of America database [6].

### DNA isolation

DNA was isolated from the whole (EDTA) blood samples using the Gentra Puregene Blood Core Kit B (Qiagen, Venlo, Netherlands) using a modified protocol as described previously [7]. DNA was isolated from hair samples using the Quick-DNA Miniprep Plus Kit (Zymo Research, Irvine, CA, USA) according to the manufacturer’s protocol and from semen (straw) using the Gentra Puregene Blood Core Kit B (Qiagen, Venlo, Netherlands) following the modified protocol described previously [8]. DNA was isolated from blood cards, tissue sampling units (TSUs), and tissue using the Gentra Puregene Blood Core Kit B (Qiagen, Venlo, Netherlands) with modifications (Additional File 2). All DNA was quantified using an Epoch Microplate Reader (BioTek, Winooski, VT, USA) and stored at -20˚C.

### SNP genotype analysis

SNP genotypes at 95,256 loci (GGP Bovine 100 K, Neogen, Lincoln, NE) were on hand for 721 individuals within the UNL system, including 6 affected calves. An initial association analysis was performed in GCTA [9] using the fast-GWA-GLMM model, which included a sparse genomic relationship matrix (GRM) to account for relationships among individuals and included the saddle point approximation (SRA) method to correct for inflated *P-*values due to an imbalance in the samples size of cases and controls. SNPs were removed if their minor allele frequency was less than 0.0001 or if fewer than 90% of individuals had genotypes for the marker. Plink 1.9 [10] was employed using the same SNP data to identify regions of homozygosity (–homozyg) shared by the affected calves.

Genotype data were computationally phased with fastPhase [11] using default parameters after creating the input file containing the chromosome 29 region of interest with Plink [10]. After identification of the candidate haplotype, SNP data were phased to identify other carriers of the haplotype to prioritize for genotyping of the candidate variant.

### Whole genome sequencing

Isolated DNA from two affected calves was sent to Admera Health (South Plainfield, NJ) for KAPA library preparation and 150 bp, paired-end sequencing on an Illumina NovaSeq to a targeted depth of 12X per animal. Raw data were pre-processed using TrimGalore [12] and reads were mapped to the ARS-UCD1.2 genome with BWA-MEM [13]. Duplicates were marked with Samtools [14]. GATK was then implemented to realign indels and call variants using GATK Haplotype Caller [15]. Variants were combined with those from 145 additional cattle previously sequenced in the lab (see Data Availability Statement for Accessions), including 35 cows from a UNL herd (Accession PRJNA1042814) with an ancestry similar to that of the affected calves.

VCFTools [16] filtered the vcf to include only biallelic markers where the two affected calves were homozygous and with a minor allele frequency across all samples less than 0.15. The resulting loci were then filtered to remove any for which more than one other animal in the sample was homozygous or for which more than two animals outside of the 37 from UNL were heterozygous. The distribution of the remaining variants across the genome was quantified and the predicted impact of each variant was determined using the Variant Effect Predictor (VEP) [17]. Sequence Read Archive (SRA) data were acquired and aligned to the ARS-UCD1.2 using the pipeline provided on the respective GitHub page [18] to generate genotypes of animals within the database for the variants with a predicted impact. Additional whole-genome sequence data from the University of Missouri, including about 5,500 cattle (Dr. Robert Schnabel, personal communication) were also queried to identify the presence and frequency of each variant in that larger dataset.

The chromosome 29 region was also visually inspected in the Integrative Genomics Viewer (IGV, Broad Inst) for any possible structural variants in *PYGM* or other loci that may not have been called due to variation in sequencing coverage.

### Genotyping of the candidate variant

Two genotyping methods were employed to assay the candidate variant, both based on PCR of the region. Genotyping was performed on all animals in the herd identified to have the candidate haplotype (*N* = 172), animals in the herd with pedigree relationship to the Red Angus suspect founder bull regardless of SNP haplotype (*N* = 71), additional animals in the herd (*N* = 98), the affected calves (*N* = 8), the suspected Red Angus founder bull (*N* = 1), progeny of the suspected founder bull (*N* = 8), and other registered Red Angus bulls (*N* = 23).

Primer3 [19] was used to design PCR primers (Additional File 3). PCR reactions were conducted using a FastStart kit (Sigma-Aldrich, St. Louis, MO, USA). The reaction mixture was comprised of 4.45 µl of MilliQ water, 0.25 µl of 25 mM magnesium chloride, 1.2 µl 10 × PCR reaction buffer with 20 mM magnesium chloride, 0.5 µl of dNTP mix, 0.1 µl of FastStart Taq DNA Polymerase, 0.75 µl of 20 µM forward and reverse primers, and 4 µl of 5 ng/µl DNA template. The thermal cycler conditions involved an initial denaturation step at 94˚C for 4 min, followed by 32 cycles of denaturation at 94˚C for 30 s, annealing at 58˚C for 30 s, and extension at 72˚C for 45 s. A final extension was performed at 72˚C for 10 min, followed by a hold at 10˚C. PCR results were visualized using gel red after electrophoresis on a 1.2% agarose gel.

For Restriction Fragment Length Polymorphism (RFLP) analysis, 8.5 µl of PCR product was combined with 0.6 µl of 50X SAM buffer, 2 µl of 10X buffer tango, 19 µl of nuclease-free water, and 1 µl of *BseMII* enzyme. The samples were incubated at 55˚ C for 1 h to activate the enzyme followed by an inactivation step at 80˚ C for 20 min. Each sample run included a non-template (negative) control, a wild-type control, and a homozygous control. The resulting product was visualized using gel red after electrophoresis on a 2% agarose gel (Additional File 4a).

Any sample failing to genotype via RFLP was genotyped by Sanger sequencing (Additional File 4b) utilizing both the forward and reverse primers (Additional File 3) performed at ACGT, INC. (Wheeling, IL, USA) after PCR cleanup, which included 0.75 µl of ExoSAP-IT (Applied Biosystems, Foster City, CA, USA) added per 3 µl of PCR product. Once ExoSAP was added, the samples were subject to thermal cycling (30 min at 37 ˚C, followed by 15 min at 80˚C, and a hold at 15˚C). The resulting sequences were analyzed using Sequencher® version 5.4.6 (Gene Codes Corporation, Ann Arbor, MI).

### Transcript analysis

After homogenization (30 mg of tissue in 300 µl of Buffer RLT with handheld homogenizer), RNA was isolated from flash-frozen *semimembranosus* muscle from Calf B, using the RNeasy Fibrous Tissue Mini Kit (Qiagen, Venlo, Netherlands) according to the manufacturer’s protocol. The RNA integrity number (RIN) and concentration were determined using a Bioanalyzer (Agilent, Santa Clara, CA). Reverse transcription for RNA ligase-mediated-rapid amplification of cDNA ends (RLM-RACE) was conducted on RNA from the *semimembranosus* muscle using the FirstChoice RLM-RACE Kit (ThermoFisher) following the 3’ RACE protocol provided by the manufacturer. The outer 3’ RLM-RACE PCR was performed with the following modified cycle: initial denaturation at 94˚C for 3 min, followed by 13 cycles of 94˚C for 30 s, annealing at a temperature starting from 64˚C and decreasing by 0.5˚C per cycle, and extension at 72˚C for 30 s. This was followed by 22 cycles of denaturation at 94˚C for 30 s, annealing at 56˚C for 30 s, extension at 72˚C for 30 s, and a final extension at 72˚C for 7 min. The reaction mixture was then held at 10˚C. The PCR product was visualized by analyzing 10 µl of the product using gel red on a 2% agarose gel.

1X AMPure XP beads (Beckman Coulter) were bound to the outer 3’ RACE PCR product at room temperature for 5 min. After creating a bead pellet with a magnetic stand, the supernatant was removed, and beads were washed twice with freshly prepared 80% ethanol. The supernatant was discarded and the beads were air dried for 5 min. The sample was incubated at room temp for 2 min after mixing with 25 µl of MilliQ water.

The sample mixture was added to the magnetic stand to pellet the beads. In a new tube, DNA was repaired by briefly mixing the supernatant, 3.5 µl of Ultra II End-Prep Reaction Buffer, and 1.5 µl of Ultra II End-Prep Enzyme Mix (New England Biolabs, Inc.). The sample was incubated at 20˚C for 30 min and then at 65˚C for 30 min in a thermal cycler with a lid set to 75˚C. Post-repair clean-up was performed by washing with 70% ethanol (described above) and eluted into 30 µl of MilliQ water.

To ligate adapters, 5 µl of NEBNext Quick T4 DNA Ligase (New England Biolabs, Inc.), 12.5 µl of Ligation Buffer, and 2.5 µl Adapter Mix (Oxford Nanopore Technologies, Kit SQK-LSK109) was added to repaired DNA. The sample was vortexed briefly before being incubated at room temperature for 15 min. Post-adapter clean-up was done by washing with 125 µl of L Fragment Buffer (as described above) and eluting into 10 µl of Elution Buffer (Oxford Nanopore Technologies).

The Flongle (Oxford Nanopore Technologies, FLO-FLG001) flow cell was primed after detecting the number of pores by removing the seal covering the spotON port and adding a mixture of 3 µl of Flush Tether and 117 µl of Flush buffer (Oxford Nanopore Technologies) by twisting the plunger of the pipette down to zero. The flow cell was loaded with a mixture containing 15 µl of Sequencing Buffer II, 10 µl of Loading Beads II (Oxford Nanopore Technologies), and 5 µl of the sample by twisting the plunger of the pipette down to zero. The flow cell was sealed, and the sample was sequenced. Passed reads were mapped to ARS-UCD1.2 using Minimap2 [20] and Samtools [14] before being visualized on IGV.

### Proteomics

At the UNL Proteomics and Metabolomics Facility (PMF), total protein was extracted from 40–60 mg of *semimembranosus* muscle from Calf B and a wild-type animal using a Thiourea/urea-based extraction method. An aliquot was reduced/alkylated before protein digestion using LysC and Trypsin. Peptides were re-dissolved in 5% acetonitrile, 0.5% formic acid, and digest was injected and run by nanoLC-MS/MS using a 2 h gradient on a Waters CSH 0.075mmx250mm C18 column feeding into a Thermo Eclipse mass spectrometer run in OT-OT mode.

All MS/MS samples were analyzed using Mascot (Matrix Science, London, UK; version 2.7.0). Mascot was set up to search the cRAP_20150130.fasta (125 entries); uniprot-refprot_UP000009136_Bos_taurus _20210923.fasta (37,513 entries); Custom12_20230405 database (sequences provided by users) assuming the digestion enzyme trypsin. Mascot was searched with a fragment ion mass tolerance of 0.060 Da and a parent ion tolerance of 15 PPM. Deamidated of asparagine and glutamine, oxidation of methionine, and carbamidomethyl of cysteine were specified in Mascot as variable modifications.

Scaffold (version Scaffold_5.2.2, Proteome Software Inc., Portland, OR) was used to validate MS/MS based peptide and protein identifications. Peptide identifications were accepted if they could be established at greater than 80.0% probability by the Peptide Prophet algorithm [21] with Scaffold delta-mass correction. Protein identifications were accepted if they could be established at greater than 99.9% probability and contained at least two identified peptides. Protein probabilities were assigned by the Protein Prophet algorithm [22]. Proteins that contained similar peptides and could not be differentiated based on MS/MS analysis alone were grouped to satisfy the principles of parsimony. Proteins sharing significant peptide evidence were grouped into clusters.

### Immunohistochemistry (IHC)

Two different antibodies were utilized for IHC. The first antibody, Thermo Scientific PA5-29,132, targeted *PYGM* (Thermo Fisher Scientific Inc. in Waltham, MA, USA). The immunogen used for this antibody was a recombinant fragment corresponding to a region within amino acids 511 and 753 of Human *PYGM*. The second antibody, AbCam anti-*PYGM* (ab231963), targeted the amino-terminal corresponding to Mouse *PYGM* amino acids 1–200 (ABCAM, Waltham, MA, USA). Ventana Benchmark Ultra staining platform (Roche Diagnostics Corporation, Indianapolis, IN, USA) was used for staining. The indicator system was alkaline phosphatase-based with Fast Red chromogen.

Myophosphorylase activity was also assessed in 8 μm frozen sections of muscle biopsies [23]. Briefly, sections were incubated in a solution containing glucose-1-phosphate, AMP, glycogen, and sucrose in a sodium acetate buffer with 95% ethanol. Sections were washed in 40% ethanol, air dried, and covered in a Gram Iodine solution. Sections mounted in glycerol with standard myophosphorylase activity stain blue, whereas myophosphorylase deficient sections stain light brown.

### Glycogen assays

Glycogen concentrations in skeletal muscle were measured by fluorometric assay as glucose residues, as described in a previous study [24]. Samples included immediately flash-frozen *semimembranosus* muscle from two affected, three carriers, and two wild-type animals. These data were analyzed across all three groups using a one-way ANOVA with a Bonferroni correction. Glycogen from five carriers, five wild-type, and one affected calf was measured from the *longissimus lumborum* muscle 24 h and two weeks after slaughter. Independent-sample t-tests were used to investigate potential differences between the wild-type and carrier groups. The affected calf was excluded from the statistical analysis of glycogen concentrations at 24 h and two weeks post-slaughter due to a sample size of one.

### Product analysis

#### Carcass data

Carcass data, including hot carcass weight, yield grade, ribeye area, marbling score, and backfat thickness, were collected from 42 wild-type and 47 carrier animals. All cattle were housed and managed in the same feedlot (UNL West Central Research and Extension Center Feedlot in North Platte, NE) and were harvested between the ages of 13.7 and 15.3 months. Independent-sample t-tests examined potential differences between the wild-type and carrier groups.

#### Sample description

*Longissimus lumborum* muscle was obtained from 10 cattle (nine steers, one heifer) that were harvested at a commercial abattoir (Tyson Foods, Lexington, NE). Before harvest the cattle were finished at the UNL West Central Research and Extension Center Feedlot (North Platte, NE), attaining an average carcass weight of 386 kg. These cattle represented two genotypes: five were wild-type for the candidate variant and the other five were carriers. The carcasses were ribbed between the 12th and 13th rib, sorted, and samples were collected at fabrication 24 h post-harvest. Day 0 was designated as the initial sampling day. One, approximately 0.635 cm slice of the 12th ribeye face was collected for 0 d expressible moisture. An approximately 15 cm section from the cranial portion of the loin was taken from the left side of each carcass, vacuum sealed, and transported on ice to the UNL Loeffel Meat Laboratory. Sample loin sections were stored in cardboard boxes at approximately 2 °C for 14 days.

Calves B and C were harvested at the UNL Loeffel Meat Laboratory under USDA inspection. Samples were prepared similarly to the commercially harvested samples, and additional sections were taken for IHC. Loin sections were removed from vacuum packaging on day 14 (Calf B) or day 9 (Calf C), deboned, and 2, 2.54 cm steaks were excised for further analysis.

#### Instrumental color

Steaks (2.54 cm) from each loin section were cut and left uncovered to bloom in the open air at approximately 5 °C on Styrofoam trays for 30 min before color evaluation. Objective color was measured through polyvinyl chloride (PVC) for wild-type and carrier animals (day 0 and day 14), Calf B (day 0 and day 14), and Calf C (day 9) using a Portable Chromameter CR-400 colorimeter (Konica Minolta, Tokyo, Japan) using the CIELAB color space (L*, a*, and b*) set with a D65 illuminant, 2 °C, and an 8 mm diameter aperture size. Three randomly positioned readings were taken from each steak and their L*, a*, and b* values were averaged for each sampling unit. The resulting values were used to calculate the a*/b* ratio, hue angle (arcTan[b*/a*]), and Chroma ([(a*^2^) + ( b*^2^)]^0.5^) for each sampling unit [25]. In the 3-dimensional CIE Lab color space, a* (x-axis) and b* (y-axis) values can be plotted, and the arc tangent of this ratio is used to describe the true color or hue of a sample. Because color becomes more vivid at the periphery of the color space, a higher a*/b* ratio and lower hue angle indicate a more vibrant color perception. Chroma is the calculation of the saturation index based on the redness and yellowness for the principal hue of the sample and can be described as the strength or dominance of the hue.

#### Cook loss

The remaining steaks from each loin section were cut to 10 cm × 7.5 cm × 2.54 cm cuboids and initial weight was recorded on day 14 for all carrier, wild-type, and affected Calf B. Steak samples were threaded at the geometric center on the transverse plane with k-type beaded thermocouple sensors to monitor temperature changes during the cooking process and were tempered uncovered to 6 ± 2 °C at room temperature on a counter. Samples were then placed on preheated (220 ± 5 °C) flat electric griddles, cooked undisturbed, and flipped at 36 ± 1 °C. When the subsequent final cooking temperature was reached (70 ± 1 °C), samples were promptly removed from the griddle, thermocouples were extracted, and sample weights were recorded. Cook loss was calculated using the following: Cook Loss% = (Δ Sample Weight/Initial Sample Weight)*100.

#### Warner–Bratzler Shear Force (WBSF)

Cooked steaks used for cook loss analysis were stored at 3 ± 1 °C overnight on PVC film-covered plastic trays. The following morning, six 1.27 cm diameter cores were obtained from each steak by hand using a cork borer parallel to the longitudinal muscle fiber’s orientation, avoiding large fat pockets or connective tissue. Subsequently, cores were sheared perpendicular to the longitudinal fiber orientation using TMS-Pro Texture Analyzer (Food Technology Corp., VA, USA) with a Warner–Bratzler shear v-notch blade attachment and a 500 N load cell with crosshead speed set at 200 mm/minute. Data for the six replications of each sample were averaged and recorded as kg of force.

#### pH

Duplicate 10 ± 0.05 g samples of each loin section were weighed and placed inside a 100 ml beaker with 90 ml of distilled, deionized H_2_O. Mixtures were homogenized for 30 s using a Polytron homogenizer (PT 2500 E, Kinematica, Switzerland) at 10,800 rpm. A stir bar was placed at the bottom of each beaker and pH was measured using a 7.0 & 4.0 pH calibrated probe (HALO pH Meter, Hanna Labs, RI, USA) while on a stir plate set at speed 6. pH of the *longissimus lumborum* muscle was taken at day 0 and day 9, by placing the probe tip into an incision made in the muscle.

#### Statistical analysis

Independent-sample t-tests were conducted to determine if there were differences between wild-type and carrier groups of cattle for all assays run. No statistical analysis was performed given the small sample size (*N* = 2) of affected calves data and it is reported in results tables as averages of duplicates or singular values.

## Results

### Clinical description

The complete blood count of the three calves with exercise intolerance (Calves A, B, and C) was within reference intervals; abnormalities noted in the serum biochemistry profiles included elevations in serum creatine kinase (CK) and aspartate transaminase (AST) activities (Table 1).

**Table 1:** Serum chemistry results from three affected calves before (Pre) and 3 h after (Post) exercise

Sample	CK (U/L)	AST (U/L)	LDH (U/L)	ALP (U/L)
Normal Range	< 350	50–100	800–1275	50–250
Calf A Pre/Post	**450**/**1092**	99/**113**	1036/1138	76/73
Calf B Pre/Post	**698**/**3711**	**104**/**155**	1253/**1519**	101/97
Calf C Pre/Post	**3213**/**5010**	-	-	**40**/83

Bolded values are outside the laboratory's established bovine normal values (Physicians Laboratory Services Inc. Lincoln NE, USA). Abbreviations: Creatine Kinase (CK), Aspartate Transaminase (AST), Lactate Dehydrogenase (LDH), Alkaline Phosphatase (ALP).

When encouraged to exercise all three calves would slow when repeatedly pushed to run, eventually becoming reluctant to move but not reaching the point of physical collapse. After the trial, they displayed mild muscle fasciculation, most noticeable in the *semimembranosus* and *semitendinosus* muscles (Additional File 5). Serum CK activity was above the reference interval at all time points and increased 1.5- to fivefold 3 h post exercise (Table 1).

**Fig. 2:**
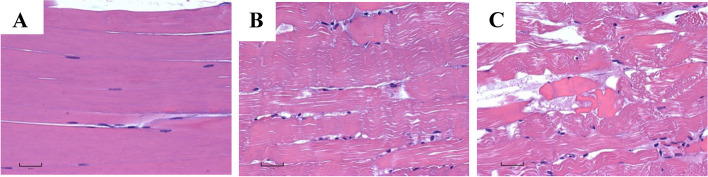
Longitudinal section of *semimembranosus* muscle (**A**-**C**) stained using hematoxylin and eosin. **A **Wild-type calf demonstrating normal muscle morphology. **B** Calf A, an affected calf, shows only mild vacuolation and fiber separation. **C** Calf B, an affected calf, shows loss of cross striations and hypereosinophilia of cytoplasm typical of myodegeneration and necrosis in addition to fiber separation

Hematoxylin and eosin-stained slides of *semimembranosus* muscle biopsy from calves A and B revealed varied eosinophilia and basophilia of muscle fibers and a slight separation of the myofibers compared to a control animal (Fig. 2A). The muscle had rare degenerate fibers with loss of striation and hypereosinophilic cytoplasm in a few surrounding macrophages (Fig. 2B-C). Calf B had occasional necrotic skeletal muscle fibers (Fig. 2C) and focal macrophage infiltration. Tissues of one previously autopsied calf were retrospectively reviewed and very rare necrotic muscle fibers were identified, sometimes surrounded by macrophages. All other organ systems were histologically normal.

**Fig. 3:**
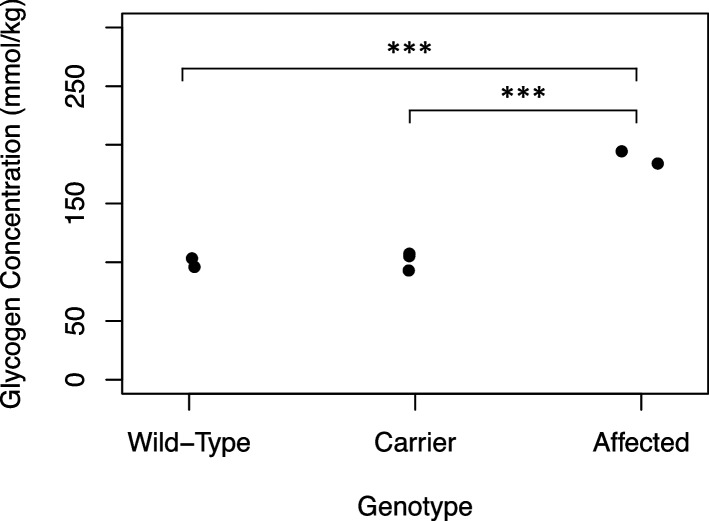
Glycogen concentrations (mmol/kg, wet weight) of *semimembranosus* muscle biopsies were measured in frozen samples of wild-type (*N* = 2), carrier (*N* = 3), and affected animals (*N* = 2). ***P < 0.001

Muscle glycogen concentrations, assayed in samples that were immediately frozen after biopsy (Fig. 3), were 1.9-fold greater (189.3 ± 7.4 mmol/kg wet weight, *N* = 2) in affected calves than in healthy herd mates (101.0 ± 6.2 mmol/kg, *N* = 5). Thus, a diagnosis of rhabdomyolysis and glycogen storage disease was established in the clinically affected composite calves.

### Pedigree analysis

A search of herd records between 2020 and 2021 identified five other calves that developed exercise intolerance when moved to new pastures. Of these calves, two were sold, two died in the pasture, and one was euthanized and necropsied after going down when being pressured to move. The 5 additional calves were related through the same, shared common ancestor on the sire side (Fig. 1). This sire was a Red Angus and Simmental composite herd bull. Thus, a cohort of 8 affected calves with a potential genetic myopathy were available for genomic analysis.

### SNP genotype analysis

**Fig. 4:**
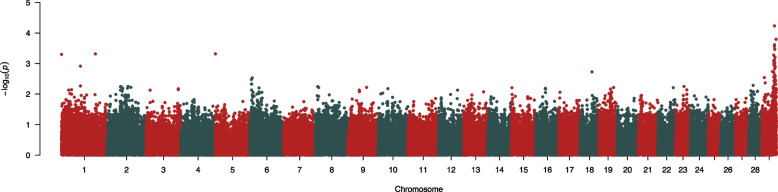
Manhattan plot after GWA in GCTA utilizing the fastGWA-GLMM method. The dataset included 721 cattle from the UNL herd (6 affected) genotyped on the Bovine 100 K GGP array. Chromosome 29 (~ 40 to 49.5 Mb) was identified as the primary region of interest

The genome-wide association analysis, including 721 cattle (6 affected) genotyped on the GGP Bovine 100 K SNP array, identified 119 significant (*P* < 0.01) markers after correction using the SRA method (Fig. 4, Additional File 6). Thirty-seven of the first 50 markers, sorted by *P-*value, were on chromosome 29. Except for three markers between 8.6 and 37.2 Mb, the other 34 variants on chromosome 29 clustered within a ~ 9.5 Mb region (40.0 to 49.5 Mb). Homozygosity analyses revealed that all six cases were homozygous for 146 loci on chromosome 29 between 42.6 and 46.2 Mb; in SNPs across this region, only 3.5 to 8.9 percent of the control cattle were homozygous for the variants present in the affected calves. Less than 25 control cattle shared homozygosity with the affected calves at SNPs between 42.6 and 43.2 Mb, identifying this as the primary region of interest. No other genomic regions were identified where all affected calves shared a homozygous genotype.

Phasing of the genotypes revealed a 36 SNP haplotype on chromosome 29 for which all affected calves were homozygous. Out of the 721 animals from the herd included in the SNP analysis, one copy of the chromosome 29 suspect haplotype was found in 183 animals, while 9 animals (including all 6 affected calves) possessed two copies of this haplotype.

### Whole-genome sequencing

Initial variant filtering across the 2 affected calves and 145 other cattle to include only biallelic positions homozygous in the affected calves and with maf < 0.15 resulted in 16,064 candidate variants. Removal of those for which one or more samples other than the affected calves were homozygous left 4,154 variants, which were then pruned down to 1,478 loci after removing those for which more than two non-herd animals were heterozygous. Based on SNP analyses, 31.5% of the remaining candidate variants were found on chromosome 29, the region of primary interest. The Ensembl Variant Effect Predictor identified 12 variants with a predicted impact on gene function. Whole-genome sequence data from the University of Missouri (*N* =  ~ 5,500) (Dr. Robert Schnabel, personal communication), and data available on the NCBI-SRA found the alternative allele of 11 of the 12 variants in other breeds than those comprising the composite, suggesting that those loci are unlikely to contain the causative variant. The remaining variant, a nonsense variant in exon 16 of *PYGM* (Chr29:g.42989581G > A), not previously reported, was predicted to result in a premature stop codon at amino acid 650 of 842 (Chr29:c.2257C > T), truncating the protein product by 33%. Due to the predicted impact of the *PYGM* variant on the protein (Chr29:p.Arg650*) and previously published identification of a *PYGM* variant in Charolais cattle with similar disease [3], this locus was prioritized for follow-up studies.

### PYGM variant genotyping

The *PYGM* candidate SNP was genotyped in 381 cattle (Table 2). All affected calves (*N* = 8) genotyped as homozygous for the variant allele (A/A). The sires (*N* = 3) and dams (*N* = 8) of the affected calves were heterozygous (G/A). The registered Red Angus sire of the herd bull was heterozygous for the *PYGM* candidate variant. As no DNA was available from his sire or dam, and all carriers with known pedigrees were descendants of this bull, he was deemed the putative founder.

**Table 2:** The count of observed *PYGM* genotypes

**Group**	**G/G**	**G/A**	**A/A**	**Total Animals**
Nebraska Herd	159	182	8	349
Red Angus Suspected Founder	0	1	0	1
Direct Progeny of the Suspected Founder	4	4	0	8
Other Red Angus	23	0	0	23
**Total**	**186**	**187**	**8**	**381**

All animals with the A/A genotype were affected. Animals from the herd were selected for genotyping based on the presence of the suspect haplotype or known relationships to affected animals and thus do not constitute a random sample of the population

Among the 381 animals genotyped for the candidate variant, 34 individuals with one copy of the associated SNP haplotype genotyped as wild-type (G/G) for the *PYGM* SNP and three animals with two copies of the SNP haplotype found in the affected calves were heterozygous for the *PYGM* SNP. The haplotype was therefore not completely predictive of the genotype at the candidate locus. In addition, three cattle of the 721 cattle with SNP genotypes initially classified as not having the suspect haplotype were heterozygous for the *PYGM* SNP. Further analysis revealed a truncated version of the candidate haplotype in each. In one individual, the haplotype was truncated by 5 SNPs (120 kb), and the two others by 657-710 kb. The portion of the candidate haplotype remining in all three included the region containing the *PYGM* SNP, showing a decay of the haplotype in these individuals.

### Transcriptomics and proteomics

RLM-RACE sequencing demonstrated transcription of *PYGM* through the candidate variant was detected in the affected calf muscle to the annotated end of the gene (Additional File 7). Label-free quantitative proteomics analysis of *semimembranosus* muscle of a wild-type animal and affected calf detected the protein product of *PYGM* only in the wild-type animal with 68% protein coverage and 302 peptide-spectrum matches, indicating high abundance in that sample.

**Fig. 5:**
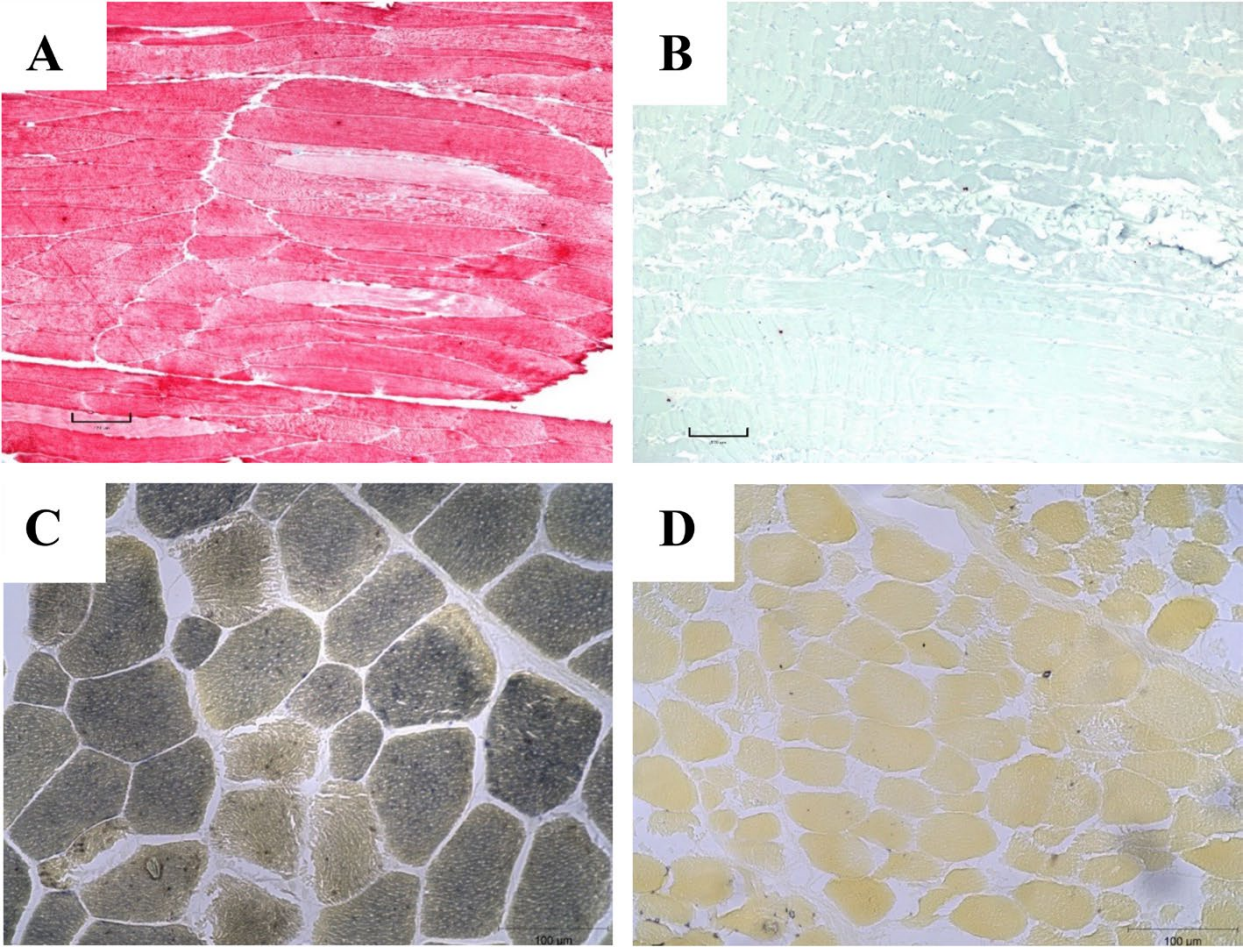
**A** Longitudinal section with immunohistochemical stain for myophosphorylase protein (red color) in a wild-type calf. **B** Longitudinal immunohistochemical stain for myophosphorylase protein in an affected calf shows no staining for myophosphorylase. **C** Histochemical stain for myophosphorylase activity in a control calf with blue staining indicating active myophosphorylase enzyme. **D** Histochemical stain for myophosphorylase activity in an affected calf with lack of staining indicating no myophosphorylase enzyme activity

IHC supported the label-free quantitative proteomics analysis, demonstrating negative *PYGM* staining in the *semimembranosus* muscle of affected calves when using antibodies targeting the *PYGM* protein (Fig. 5B). The wild-type animal exhibited positive staining, indicated by red pigmentation (Fig. 5A). Myophosphorylase activity in the affected calves was reduced (Fig. 5D) compared to the control (Fig. 5C). PAS staining did not demonstrate accumulated glycogen in the *semimembranosus* muscle of the three affected calves studied. IHC staining for *PYGM* in two heterozygous animals was not distinguishable from that of the wild-type controls.

#### Product quality measures

**Fig. 6:**
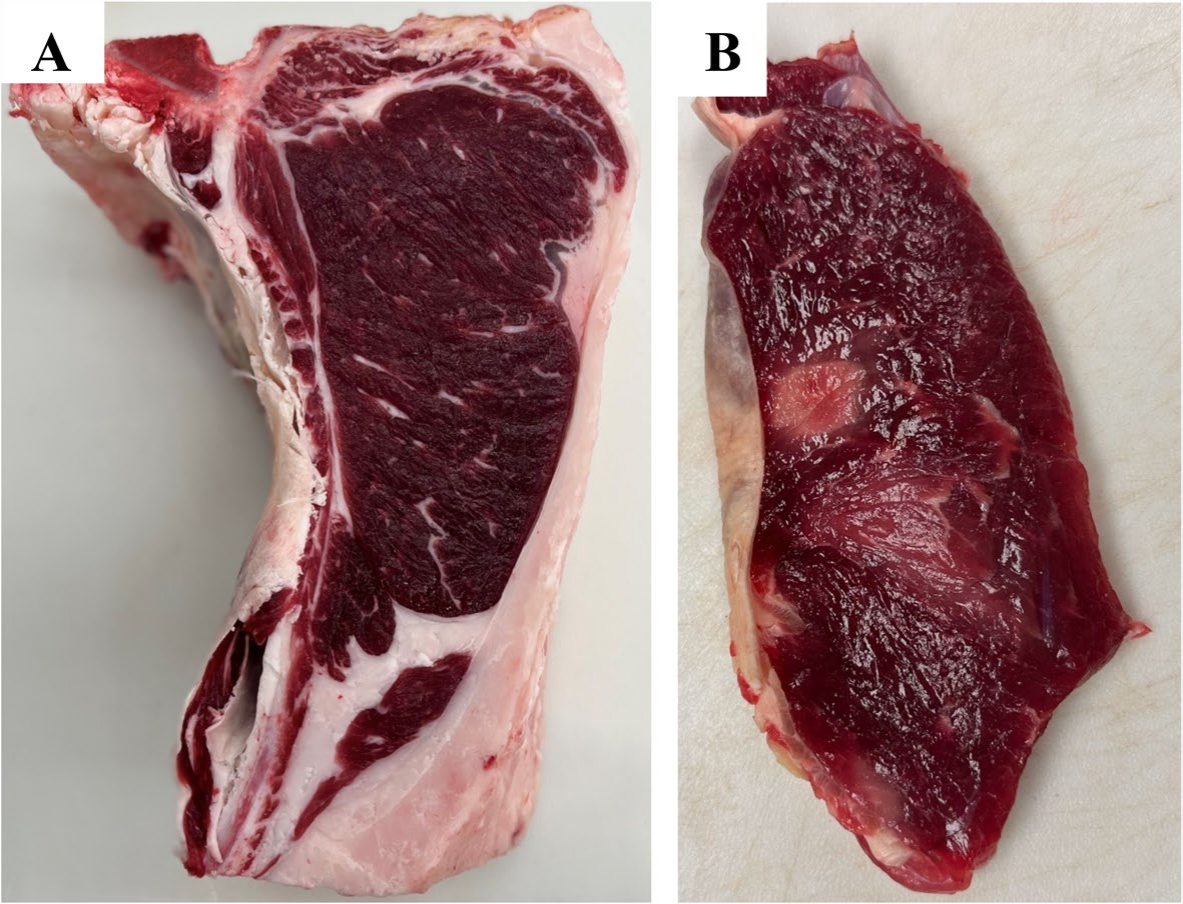
**A** *Longissimus lumborum* muscle from Calf B 24 h after slaughter. **B** *Triceps* muscle from Calf C 9 days after slaughter

**Fig. 7:**
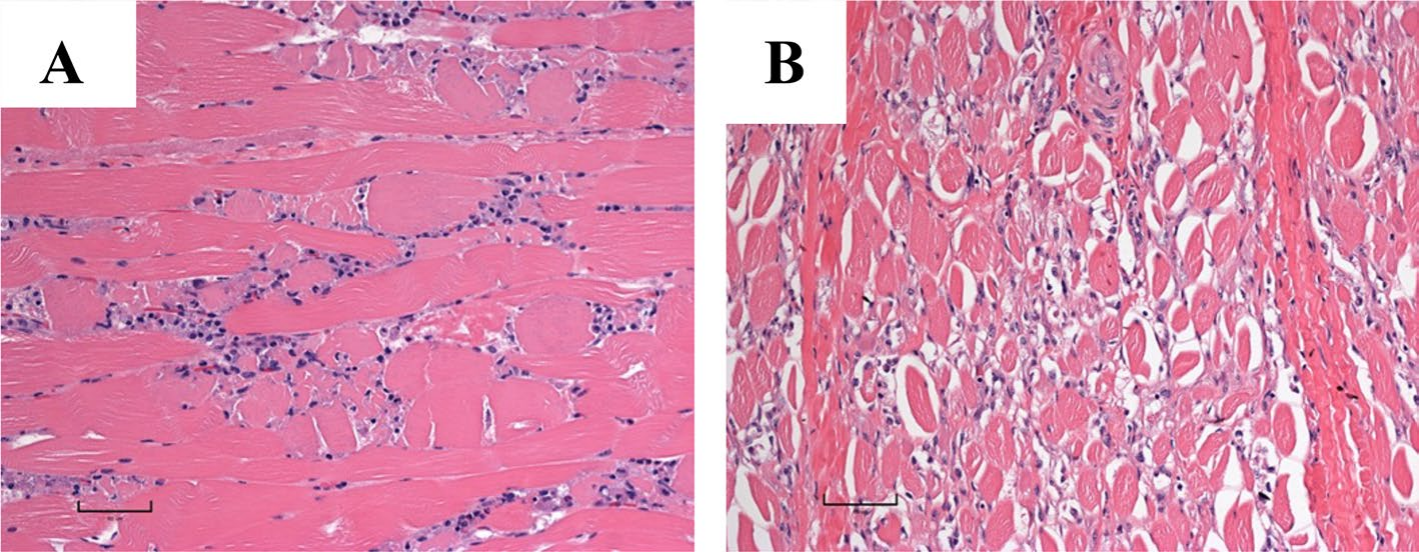
**A** Longitudinal section through *longissimus lumborum* with necrosis with macrophage infiltrates. **B** *Longissimus lumborum* with extensive fibrosis in the area of past necrosis

Following typical postmortem aging of carcasses (Calf B and Calf C), abnormalities were confined to the darkening of the skeletal muscles, presenting as dark cutters (Fig. 6A). Calf C had remarkable alternating dark and pale regions observed in the muscle (Fig. 6B), exhibiting widespread necrosis and macrophage infiltrates in the pale muscle regions (Fig. 7A). There was also marked fibrosis in these areas of pale, firm muscle (Fig. 7B). This dramatic pathology was distinct from the more typical dark cutter lesions, which lacked dramatic histologic change.

No significant differences between wild-type and carrier animals were observed in hot carcass weight, yield grade, ribeye area, marbling score, and backfat thickness. Regarding sensory-predictive characteristics such as cook loss and Warner–Bratzler Shear Force (WBSF), no significant differences were observed among wild-type and carrier animals (Additional File 8 and Additional File 9). The instrumental color values for lightness (L*), redness (a*), and yellowness (b*) were also similar among the groups. However, there were differences (*P* < 0.05) in hue angle, a/b ratio, and pH (Additional File 10). Product from carriers of the *PYGM* variant had a more vivid and vibrant red color (22.58) than that from the wild-type group (24.11), as indicated by the hue angle. Wild-type animals had a greater pH (5.53) than carriers (5.48), but both fell within the normal range of pH. There were no notable differences in chroma between wild-type and carrier samples. Due to a limited sample size, meat quality characteristics were not included in the statistical analysis for the affected calves (Additional File 11 and Additional File 12). Calf B and C represented dark-cutting beef characteristics, including higher ultimate pH, darker color (lower L* values), and less redness (lower a*values) than found in normal-colored beef. The extensive fibrosis seen histologically and palpable firmness of the light focal lesions negatively impact meat quality and would likely result in extensive trim or loss of carcass value at slaughter.

There was no significant difference in glycogen concentrations in the *longissimus lumborum* muscle when comparing wild-type (24.2 ± 6.2 mmol/kg, *N* = 5) and carrier (35.2 ± 12.6 mmol/kg, *N* = 5) animals as quantified 24 h after harvest. Two weeks after harvest, samples from animals with the wild-type genotype exhibited significantly (*P* = 0.01) greater glycogen concentrations (49.8 ± 9.9 mmol/kg, *N* = 5) compared to the carriers (30.6 ± 3.1 mmol/kg, *N* = 5). Due to the limited sample size, glycogen concentrations from LD muscle were not included in the statistical analysis for Calf B.

## Discussion

Clinical and genetic analyses of the cattle confirmed the presence of Glycogen Storage Disease Type V (GSD-V) associated with a nonsense variant in *PYGM* in these composite calves attributed to a variant distinct from that previously identified in Charolais cattle [3]. This genetic variant resides in the C-terminal domain, where a lysine residue at position 681 forms a covalent bond with the cofactor pyridoxal 5’-phosphate [26]. Pyridoxal 5’-phosphate is a cofactor essential for myophosphorylase activity, facilitating catalytic processes by directly engaging in noncovalent interactions with either the phosphate substrate or the phosphate group of the glucose 1-phosphate product [27]. Although *PYGM* was found to be transcribed in the affected calves, proteomic analyses failed to detect the protein product, demonstrating putative nonsense-mediated decay. Myophosphorylase, the protein product of *PYGM*, plays a vital role in glycogenolysis by converting glycogen into glucose-1-phosphate [28]. The inability to metabolize glycogen results in an energy deficit of substrates such as ATP for muscle contraction, resulting in exercise intolerance and muscle degeneration [29]. Rhabdomyolysis in affected calves was evident based on abnormal elevation in serum creatine kinase activity, which is muscle specific [30], and in mild elevations in aspartate transferase (AST) and lactate dehydrogenase (LD) activities. Histology on muscle samples from three affected calves confirmed the presence of rhabdomyolysis with evident acute degenerative changes, separation of myofibers, myocyte necrosis, and macrophage infiltration. Fibrosis was pronounced in severely affected muscles. Glycogen accumulated in the muscle of *PYGM*-deficient calves to concentrations almost two-fold higher than the 80–100 mmol/kg glycogen concentrations in a wild-type or carrier animal [31].

The inability to break down glycogen had detrimental consequences not only on animal welfare, which was the first observed indication of an issue, but also on the meat quality of affected cattle that reached harvest. The efficient utilization of stored glycogen in post-mortem processes is crucial to producing high-quality beef [32]. Following slaughter, anaerobic metabolism continues, metabolizing glycogen in the glycolytic pathway. As a consequence of anaerobic glycolysis, lactic acid is produced [33], decreasing the meat pH from approximately 7.1 to between 5.4 and 5.7 [34]. The absence of myophosphorylase inhibits glycogenolysis, limiting lactic acid production from glycogen reserves. Consequently, affected calves were classified as dark cutters, characterized by dark-red lean that may have a purplish hue instead of the desired bright cherry-red color. This negatively impacts the consumer’s perception of the product, reduces shelf life, and results in economic loss. The association of this variant with dark cutting beef is unique as prior work to identify genomic loci of major effect contributing to the phenotype was largely unsuccessful [35]. The current result undoubtedly benefitted from the study of a single bloodline, but together with Lei et al. [35], provides further evidence that genomic studies of dark cutting beef are warranted. With respect to animals carrying the variant, although the a*/b* ratio, hue angle, and pH were statistically between those and wild-type animals, those criteria are not expected to result in practical disparities in meat quality. The significant difference in glycogen concentration observed after two weeks in the carrier cattle compared to the control was also not expected to impact meat quality.

Nonsense-mediated decay (NMD) halts the translation of transcripts containing a premature stop codon followed by at least one intron to prevent the accumulation of faulty proteins [36]. The variant identified in exon 16 of 20 in *PYGM* strongly indicates that the absence of this protein may be attributed to NMD. This inference is further supported by the variant’s location, which is > 50 nucleotides upstream of an exon-junction complex [37]. The NMD process involves the transcription of the sequence into an mRNA transcript, as evidenced by the RACE product, followed by degradation during translation [36]. The results from the proteomic analysis and IHC align with the hypothesis of NMD due to the absence of the PYGM protein product in the affected calves, which was present in the skeletal muscle of wild-type animals.

A comparable disease has been described in Merino sheep and humans. In sheep, the condition was classified as GSD-V, and presented similarly to the calves in this study; the sheep demonstrated exercise intolerance, a lack of myophosphorylase, and excess glycogen attributed to a splice-site mutation in *PYGM* [38]. In humans, there are 147 pathogenic variants and 39 polymorphisms related to an autosomal-recessive disorder in *PYGM* termed McArdle’s disease [26]. Like the affected cattle, affected humans experience muscle fatigue and weakness during exercise and may have difficulty performing tasks requiring prolonged exertion [39]. Strategies to manage McArdle’s disease in humans consist of regulating the intensity of physical activity and providing alternated energy substrates such as fatty acids or circulating blood glucose [40]. Adjusting nutrition and exercise enables individuals with McArdle’s disease to lead relatively normal lifestyles. However, compared to monogastric humans, applying similar management strategies would likely not be achievable or practical for ruminants in a production environment and the degradation of product negates the economic incentive to do so.

In summary, the recessive *PYGM* variant identified in these cattle results in GSD-V and negatively affects muscle metabolism. This condition poses a welfare concern for affected animals and has a negative economic implication due to its effect on animal morbidity and mortality and the subsequent meat quality of affected animals that make it to harvest. While these data provide no evidence of a negative phenotype in carriers of this variant, it is crucial to identify them in breeding herds to prevent the production of affected calves.

## Conclusions

This study provides evidence that myophosphorylase deficiency in these Red Angus composite cattle is caused by a nonsense variant in *PYGM*. This genetic anomaly significantly disrupts muscle metabolism, giving rise to welfare concerns in affected animals and detrimentally influencing the economic aspects of raising livestock. The repercussions extend to the survival of the animals and subsequently have adverse effects on the quality of the meat they produce when they reach harvest. While the phenomenon of dark-cutting beef is not novel, the understanding of the genetic factors at play is limited. This study stands out as one of the first to pinpoint a specific genetic variant linked to this condition, advocating for future research into the genetics of dark cutting beef. Although carriers of this variant may not affect the beef industry immediately, identifying them within breeding herds is imperative. This proactive measure is crucial for preventing the production of affected calves, ensuring the sustained well-being of the animals and upholding quality standards of the final beef product.

### Supplementary Information

**Supplementary Material 1.**

**Supplementary Material 2.**

**Supplementary Material 3.**

**Supplementary Material 4.**

**Supplementary Material 5.**

**Supplementary Material 6.**

**Supplementary Material 7.**

**Supplementary Material 8.**

**Supplementary Material 9.**

**Supplementary Material 10.**

**Supplementary Material 11.**

**Supplementary Material 12.**

## Data Availability

Whole-genome sequence data generated for this project are available in the NCBI SRA under the BioProject PRJNA1026143 to become public upon acceptance (reviewer link: https://dataview.ncbi.nlm.nih.gov/object/PRJNA1026143?reviewer=8qiu5vv1muhr55bg dunq0bges4). Whole-genome sequence utilized as control data are available under accessions PRJNA1042650, PRJNA1042814, PRJNA513064, PRJNA663547, PRJNA1013498, and PRJNA994471. Sequence data from 20 animals are not publicly available due to ongoing projects or confidentiality agreements but are available upon reasonable request to the authors. 100 K SNP genotype data utilized for GWA and haplotype analyses are available on the Open Science Framework (Petersen, Jessica L. 2023. “An Autosomal Recessive Mutation in PYGM Causes Myophosphorylase Deficiency in Red Angus Composite Cattle.” OSF. November 29. osf.io/6whtj); cattle are identified with International Committee for Animal Recording (ICAR) identifiers and are recorded via SimGenetics (herdbook.org/).
