# Supplementary material for: An autosomal recessive variant in PYGM causes myophosphorylase deficiency in Red Angus composite cattle
Source: BMC Genomics. 2024 Apr 27;25:417. doi: 10.1186/s12864-024-10330-1 (PMC11055281; doi:10.1186/s12864-024-10330-1)
Supplement: Supplementary file 2 — Supplementary Material 2. [file 12864_2024_10330_MOESM2_ESM.docx]

**Additional File 2. Supplemental DNA Isolation Methods**

**Blood Cards:**

Two 6 mm punches were combined with 900 µl of red blood cell (RBC) lysis buffer and incubated while shaking for 5 minutes at 22˚C. The samples were centrifuged at 13,000 x g for 2 minutes, and the supernatant was discarded. The residual pellet was incubated with 450 µl of RBC lysis buffer while shaking for 5 minutes at 22˚C. The samples were centrifuged again at 13,000 x g for 2 minutes, and the supernatant was discarded. The pellet was resuspended in 900 µl of cell lysis buffer and 6 µl of proteinase K, vortexed, and incubated for 40 minutes at 35˚C. After incubation, the punches were removed, and the samples were cooled to room temperature on ice. Next, 200 µl of protein precipitation solution was added, the samples were vortexed, and incubated (5 minutes on ice). The samples were centrifuged at 13,000 x g for 2 minutes to generate the protein pellet. The supernatant was transferred to a new tube containing 800 µl of 100% isopropanol and 1 µl of glycogen (20 mg/mL) and inverted 50 times to precipitate the DNA. The samples were centrifuged at 8,000 x g for 2 minutes to pellet the DNA. The supernatant was discarded and the DNA pellet dried for 1 minute before washing with 300 µl of 70% ethanol and centrifuging at 8,000 x g for 1 minute. The supernatant was discarded and the pellet dried for 15 minutes. Finally, the DNA pellet was rehydrated in 50 µl of DNA hydration solution and incubated at room temperature overnight.

**Tissue Sampling Units (TSUs):**

225 µl of cell lysis buffer and 7 µl of proteinase K were added to 75 µl of TSU liquid sample and incubated for 1 hour at 55˚C. Following incubation, the samples were cooled to room temperature on ice. Each sample was combined with 100 µl of protein precipitation solution, vortexed for 20 seconds, and then incubated on ice for 5 minutes. The samples were centrifuged at 15,000 x g for 5 minutes at 15˚C and the supernatant was poured into a new tube containing 650 µl of 100% isopropanol. The mixture was inverted 50 times to precipitate the DNA. The samples were centrifuged at 15,000 x g for 1 minute to pellet the DNA. The supernatant was discarded and the DNA pellet was dried for 1 minute. The DNA pellet was washed with 300 µl of 70% ethanol and centrifuged at 15,000 x g for 1 minute. The pellet was dried for 1 minute. The DNA pellet was rehydrated with 75 µl of DNA hydration solution, incubated for 5 minutes at 55˚C, and left at room temperature overnight.

**Tissue:**

40 mg of tissue was combined with 300 µl of cell lysis buffer and 7 µl of proteinase K and incubated overnight at 55˚ C. After incubation, the samples were cooled on ice (1 minute) before adding 100 µl of protein precipitation solution. The samples were vortexed and incubated on ice for 5 minutes. The samples were then centrifuged at 15,000 x g for 5 minutes at 15˚ C and the supernatant was transferred into a new tube containing 650 µl of 100% ethanol. The mixture was inverted 50 times to precipitate the DNA. The samples were centrifuged at 15,000 x g for 1 minute. The supernatant was discarded and the DNA was set out to air dry for 1 minute. The DNA pellet was washed with 300 µl of 70% ethanol and centrifuged at 15,000 x g for 1 minute. The supernatant was discarded and the DNA pellet was set out to air dry for 5 minutes. Finally, the DNA pellet was rehydrated with 200 µl of DNA hydration solution, incubated at 55˚ C for 5 minutes and left at room temperature overnight.
