# Supplementary material for: An autosomal recessive variant in PYGM causes myophosphorylase deficiency in Red Angus composite cattle
Source: BMC Genomics. 2024 Apr 27;25:417. doi: 10.1186/s12864-024-10330-1 (PMC11055281; doi:10.1186/s12864-024-10330-1)
Supplement: Supplementary file 4 — Supplementary Material 4. [file 12864_2024_10330_MOESM4_ESM.pdf]

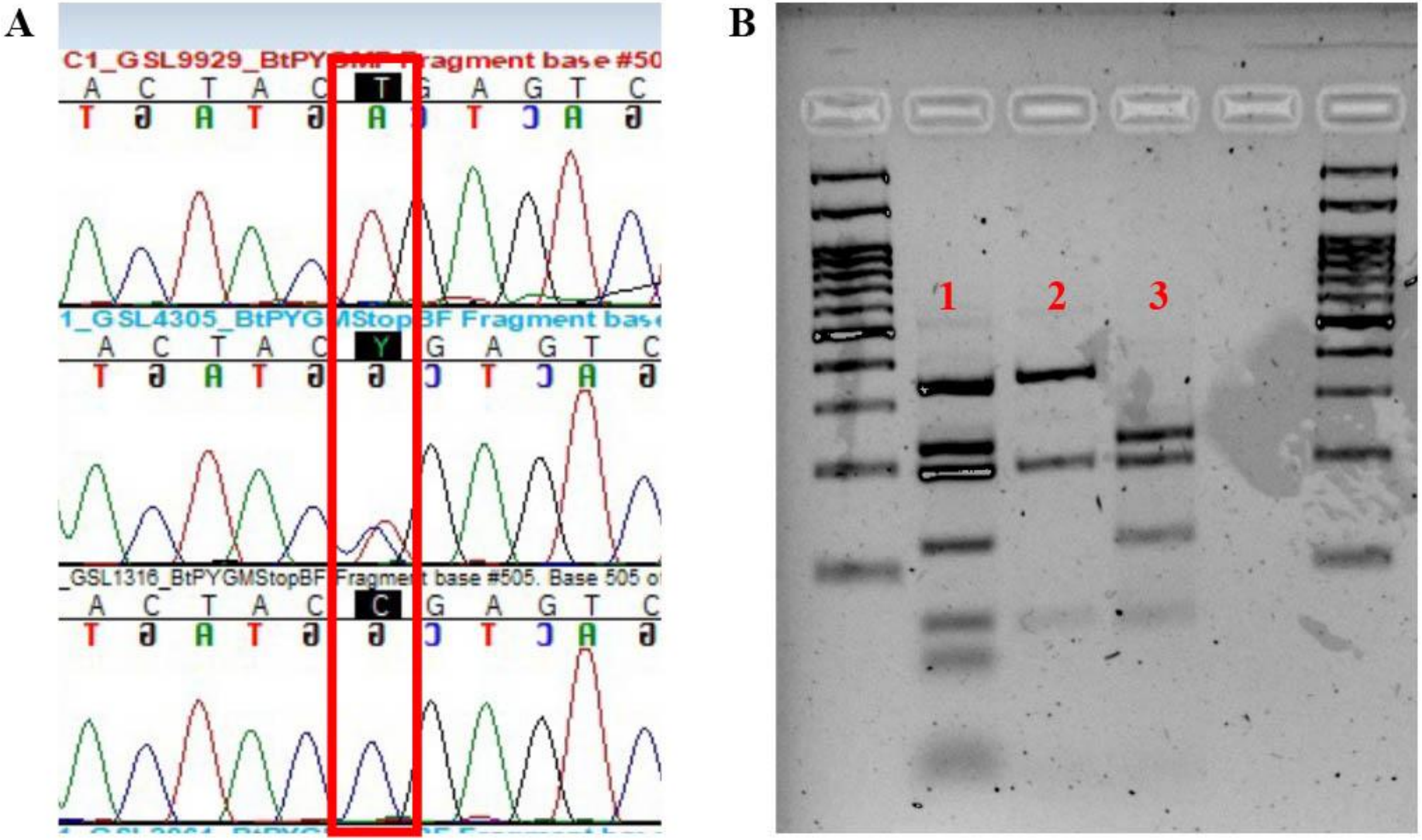

Additional File 4. **Cattle were genotyped for the *PYGM* variant using one of two methods, by Sanger sequencing or PCR-RFLP.** (A) Sanger sequencing chromatogram of an affected calf (A), a heterozygous carrier of the *PYGM* variant (B), and a wild-type control (C); (B) Agarose gel electrophoresis of PCR-RFLP product including a heterozygous carrier (lane 1), wild-type control (lane 2), and affected calf (lane 3).
