# Supplementary material for: An autosomal recessive variant in PYGM causes myophosphorylase deficiency in Red Angus composite cattle
Source: BMC Genomics. 2024 Apr 27;25:417. doi: 10.1186/s12864-024-10330-1 (PMC11055281; doi:10.1186/s12864-024-10330-1)
Supplement: Supplementary file 6 — Supplementary Material 6. [file 12864_2024_10330_MOESM6_ESM.pdf]

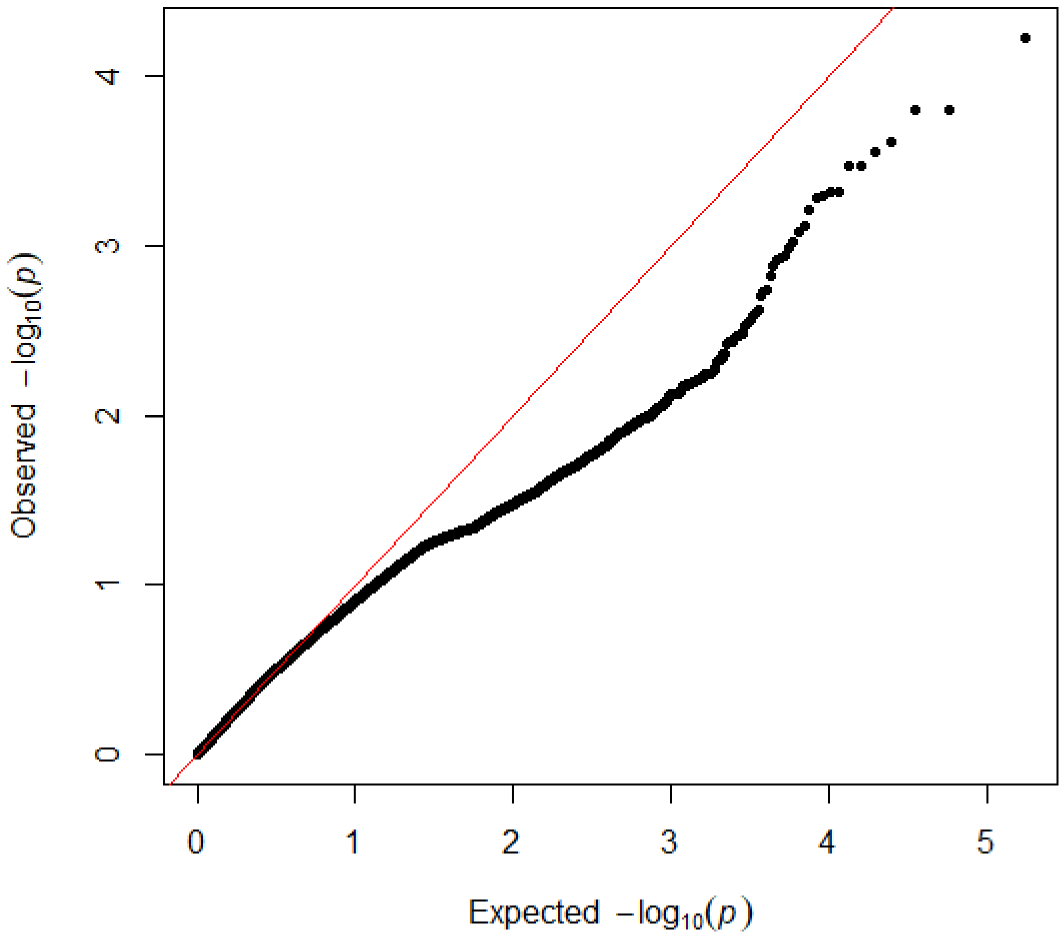

Additional File 6. Quantile-quantile (QQ) plot from the GWAS conducted with 100K SNP data on 721 individuals (6 cases, 715 herdmates).
