# Supplementary material for: An autosomal recessive variant in PYGM causes myophosphorylase deficiency in Red Angus composite cattle
Source: BMC Genomics. 2024 Apr 27;25:417. doi: 10.1186/s12864-024-10330-1 (PMC11055281; doi:10.1186/s12864-024-10330-1)
Supplement: Supplementary file 7 — Supplementary Material 7. [file 12864_2024_10330_MOESM7_ESM.pdf]

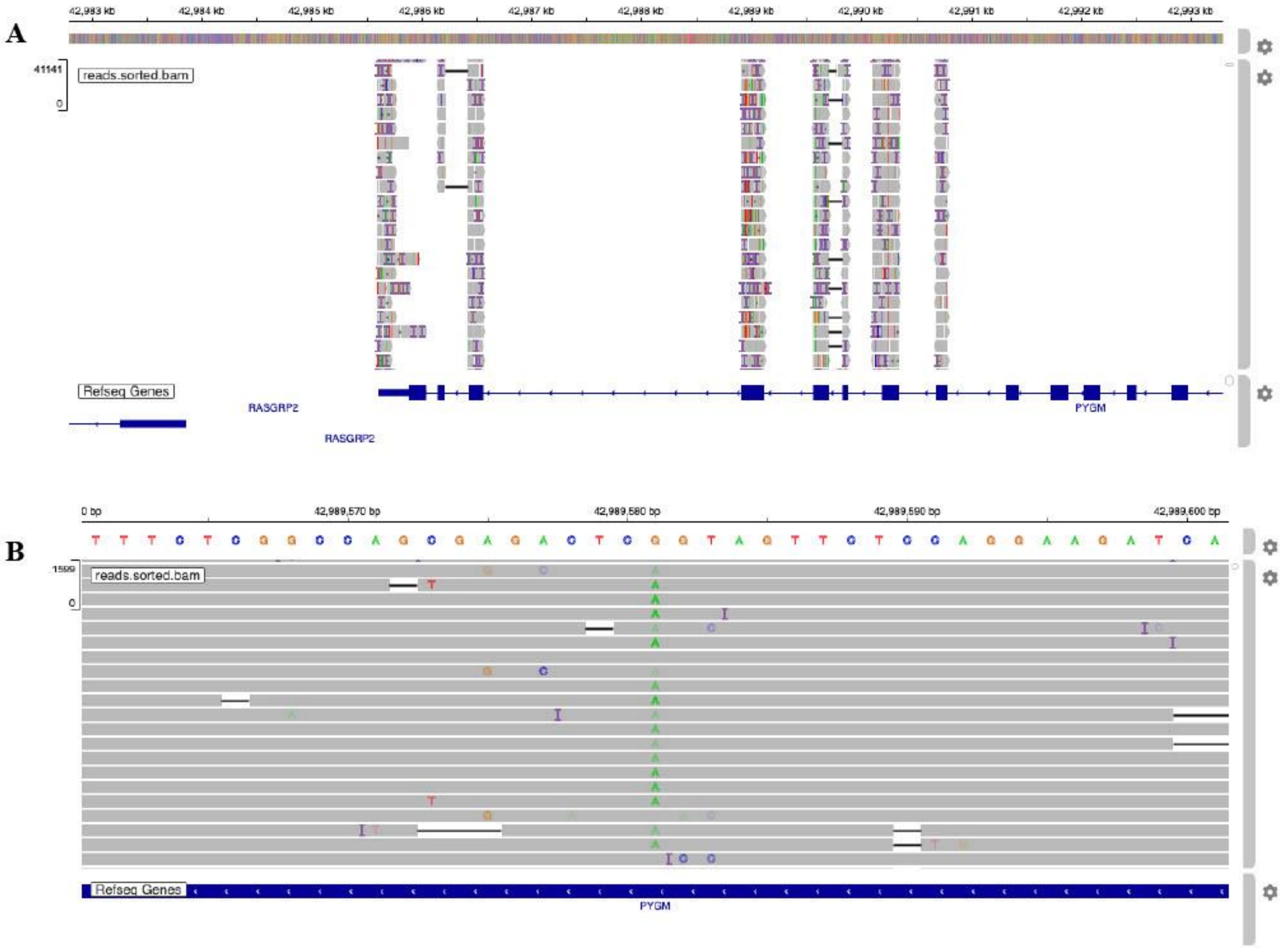

Additional File 7. Nanopore/Flongle sequencing of RNA, imaged in the Integrated Genome Viewer after 3' RACE of *semimembranosus* muscle of an affected calf. The 5' primer is placed in Exon 13 of the gene, which codes on the reverse strand. Results demonstrate the transcript terminates at the annotated 3' end of the gene (A) and contains the candidate variant (B).
